# Biosorption potential of natural, pyrolysed and acid-assisted pyrolysed sugarcane bagasse for the removal of lead from contaminated water

**DOI:** 10.7717/peerj.5672

**Published:** 2018-09-28

**Authors:** Ghulam Mustafa Shah, Muhammad Nasir, Muhammad Imran, Hafiz Faiq Bakhat, Faiz Rabbani, Muhammad Sajjad, Abu Bakr Umer Farooq, Sajjad Ahmad, Lifen Song

**Affiliations:** 1 Department of Environmental Sciences, COMSATS University Islamabad, Vehari Campus, Vehari, Punjab, Pakistan; 2 Yantai Institute, China Agricultural University, Yantai, Shandong, China

**Keywords:** Sugarcane baggasse, Equilibrium modelling, Wastewater treatment, Waste reutilization, Lead, Biosorption

## Abstract

Lead (Pb) is a ubiquitous pollutant which poses serious threats to plants, animals and humans once entered into the food chain *via* contaminated industrial effluents on their discharge into the surface of water bodies and/or geological materials. This study aimed to examine and compare the biosorption potential of natural sugarcane bagasse (NB), pyrolysed sugarcane bagasse (PB) and acid assisted pyrolysed sugarcane bagasse (APB) for the removal of Pb from contaminated water. To explore this objective, a series of batch experiments were conducted at various adsorbent mass (0.25, 0.5, 0.75, 1.0 g per 100 ml contaminated water), initial Pb concentration (7, 15, 30, 60 and 120 ppm), and contact time (7, 15, 30, 60 and 120 min). Results revealed that all the tested bio-sorbents have potential to adsorb and remove Pb ions from the contaminated water. In this regard, APB proved more effective since it removed 98% of Pb from aqueous solution at initial Pb concentration of 7 ppm and mass of 0.25 g per 100 ml of aqueous solution. The respective values in case of NB and PB were 90 and 95%. For a given adsorbent type, Pb adsorption decreased by increasing the mass from 0.25 to 1.0 g per 100 ml of aqueous solution. However, the greatest Pb removal occurred at adsorbent mass of 1.0 g per 100 ml of aqueous solution. Initial Pb concentration had a great impact on Pb adsorption and removal by adsorbent. The former increased and the latter decreased with the increase in initial Pb concentration from seven to 120 ppm. At seven ppm Pb concentration, maximum Pb removal took place irrespective to the adsorbent type. Out of the total Pb adsorption and removal, maximum contribution occurred within 15 min of contact time between the adsorbate and adsorbent, which slightly increased till 30 min, thereafter, it reached to equilibrium. Application of equilibrium isotherm models revealed that our results were better fitted with Freundlich adsorption isotherm model. Overall, and for the reasons detailed above, it is concluded that sugarcane bagasse has capabilities to adsorb and remove Pb ions from contaminated water. Its bio-sorption potential was considerably increased after pyrolysis and acid treatment.

## Introduction

Good quality water is essential for an organism’s life and is used for agricultural, industrial and domestic purposes (Renge, Khedkar & Pande, 2012). However, these agricultural, industrial and domestic activities are polluting freshwater bodies day by day. Varieties of pollutants from these sources are continuously discharged into the water system and thus adversely affect the ecosystem. Among these, water contamination by heavy metals has become one of the major concerns. Many activities of metal plating, petroleum refining, glass production, tanneries, pesticides, and battery manufacturing are producing heavy metals, i.e., lead (Pb), mercury, copper, zinc, cadmium, nickel and chromium (Kadirvelu, Thamaraiselvi & Namasivayam, 2001). Pb is a ubiquitous pollutant, and its contamination has become worldwide concern owing to its serious environmental implications. Besides, it is highly toxic and ranked at second position among the hazardous substances known to date in America (ATSDR, 2008). Its main sources in the wastewater include the industrial effluents especially from radiator manufacturing, storage batteries industries, mining operations, metal plating, tanneries, smelting, chloralkali, and alloy industries (Kadirvelu, Thamaraiselvi & Namasivayam, 2001). Additionally, the aqueous wastes of manufacturing process like television tube, paints, pigments, fuel, matches and explosives result in contamination of the wastewater (Ahalya, Kanamadi & Ramachandra, 2005). When this wastewater is exposed to the ecosystems, the metal ions transfer and accumulate into the human body either directly through intake or through the food chain (Abdel Salam, Makki & Abdelaal, 2011). Continuous accumulation of Pb in the body results in malfunction of organs and chronic toxicity. Moreover, Pb affects kidney, central nervous system and reproductive system (Badmus, Audu & Anyata, 2007).

Several attempts have been and are being made to remove Pb from contaminated water. Among them, the most common include, ion exchange (Dabrowski et al., 2004), chemical precipitation (Charerntanyarak, 1999), electrolysis, electro-coagulation (Milhajlovic et al., 2015), solvent extraction, and membrane separation (Bentama et al., 2004). However, the aforementioned methods are either expensive, expert-oriented, generate abundant toxic sludge and are limited to certain concentration of metal ions (Abas et al., 2013). Adsorption, on the other hand, has been proven to be very effective technique for remediation of heavy metals contaminated wastewater streams. It is an effective purification and separation technique in which the metal ions from contaminated water are adsorbed on surface of an adsorbent and thereby can be removed from wastewater stream (Milhajlovic et al., 2015). In this phenomenon, adsorbents that are of low-cost and have greater adsorption potentials are used. A variety of local materials, including natural, industrial and agricultural wastes, can be used for this purpose (Atkinson, Bux & Kasan, 1998). Literature revealed that the activated carbon has been extensively used for remediation of Pb contaminated wastewater (Huang & Wu, 1975). However, because of its extensive use in wastewater treatment industries, it remains an expensive material (Cheremisinoff & Morresi, 1978). Thus, more efficient, economically viable and environment-friendly adsorbent is needed for this purpose. Recently, researchers investigated the adsorption potential of orange peels (El-Said, Gamal & Mansour, 2012), cocoa shells (Meunier et al., 2003), banana peels (Annadurai, Juang & Lee, 2002), rice husks (Asrari, Tavallali & Hagshenus, 2010), pinus pinaster bark (Vasconcelos & Beca, 2006), egg shell (Hussain & Shariff, 2014), saponified melon peels (Chaudhary & Ijaz, 2014) and bentonite (Naseem & Tahir, 2001) for Pb removal from contaminated water. Besides the preparation of adsorbents, various attempts have been made to enhance the adsorption capacities of the material through physical, chemical and biological ways (Kumar & Bandyopadhyay, 2006).

In the present study, we investigated the adsorption potential of: (i) natural sugarcane bagasse (SB) obtained from the sugarcane juice extraction plants and (ii) modified SB through pyrolysis and chemically impregnation. SB possess porous character with high ion exchange capacity, contain complex mixture of polymeric functional groups and have high, cellulose, hemi cellulosic material, pectin, lignin and small amount of protein (Demirbas, 2008; Kocasoy & Guvener, 2009). These characteristics can make it a very good biosorbent. Additionally, SB exist in abundance, is economical, and very effective material. According to Chaudhary & Ijaz (2014), a low cost adsorbent should have the following benefits: effectiveness with high selectivity, simple synthesis operation, large adsorption capacity, abundantly available material. These all advantages can be achieved by using the SB. Studies investigating the biosorption potential of SB for Pb are scarce. Of the work done so far on SB, focus remained on evaluating the biosorption potential of natural SB (Putra et al., 2014; Palin Jr et al., 2016) and its chemical modifications with sulphuric acid (Martín-Lara et al., 2010) and acrylic acid (Kong et al., 2014). Further, SB was biologically modified after colonization by *Pleurotus ostreatus*, *Lentinula edodes*, *Pleurotus ostreatus* and *Ganoderma lucidum* (Palin Jr et al., 2016; Anastopoulos et al., 2017). However, to the best of our knowledge sorption potential of pyrolysed and acid assisted pyrolysed SB has not been investigated so far. Therefore, the present study aims: (i) to evaluate and compare Pb biosorption potential of NB, PB and APB and (2) to estimate biosorption behavior of natural and modified SB under varying adsorbate and adsorbent concentrations.

## Material and Methods

### Preparation of adsorbents

Sugarcane bagasse was taken from a nearby sugarcane juice plant. The material was manually chopped and later washed four times with distilled water in order to remove impurities and extra sugar contents. Thereafter, it was oven-dried at 40 °C for 48 h, and shredded in a grinding machine to pass through a one mm sieve. This natural (raw) sugarcane bagasse was named as NB.

The natural oven-dried sugarcane bagasse was placed in a pyrex flask connected with glass rod forming a bended outlet for removal of vapor and gases from the muffle furnace. Moreover, junction of glass rod and the flask was sealed with high temperature resistant silicon grease to avoid entry of oxygen to the working area (Naeem et al., 2017). Inside the muffle furnace, the temperature increase was set at a rate of 8–9 °C min^−1^. Further, temperature of 300 °C was maintained for 20 min. After that, the muffle furnace was allowed to cool down, until the temperature of reaction chamber reached down to 40–50 °C. After cooling, the pyrex flask was removed from reaction chamber of muffle furnace and biochar was collected. It was named as pyrolysed sugarcane bagasse (PB).

In another scenario, the natural oven-dried sugarcane bagasse was treated with phosphoric acid (H_3_PO_4_, 20%) solution for two hours with impregnation ratio of 2:1 (H_3_PO_4_:Sugarcane bagasse, v/w). The impregnated mixture of H_3_PO_4_ and SB was ball milled, heat treated in muffle furnace at 400 °C for 20 min, and washed with hydrochloric acid (10%) as done by Chen et al. (2012). Thereafter, the material was washed with hot distilled water thrice and subsequently dried in an oven at 105 °C for 24 h. It was named as acid-assisted pyrolysed sugarcane bagasse (APB).

### Characterization of adsorbents

Representative samples of all the three adsorbents were characterized using Fourier transform infrared spectra **(**FTIR) and scanning electron microscopy (SEM) techniques. Matson Polaris IR spectra of the adsorbent FTIR spectrophotometer was used at room temperature for the determination of IR spectra. The later was obtained in absorbance mode by pressing the particles sample with KI or KBr powder to form pellets. For each IR spectrum, a resolution of two cm^−1^ and a total of 96 scans were collected. For the study of surface morphological characteristics of the adsorbents, scanning electron microscope of high magnification power typically at five and 10 kV at 20 mm distance was used. The adsorbent’s samples were coated with gold using a Blazer’s Spluttering device before running through SEM.

### Preparation of synthetic wastewater

Synthetic wastewater of Pb was prepared using lead nitrate Pb(NO_3_)_2_ salt. Stock solution of 1,000 ppm Pb was prepared in 1,000 ml flask by dissolving 1.59 g of Pb (NO_3_)_2_ in one L of distilled water. From this, different sub-stocks of Pb concentration: 7, 15, 30, 60 and 120 ppm were prepared in separate flasks by the dilution of stock solution. Few drops of 1M NaOH/HCl solution were added in sub stock solutions to adjust the pH at 6–6.5. At this level of pH, maximum adsorption for divalent metal can be attained (Ahmad et al., 2017).

### Batch experiment

A series of batch experiment was run in the laboratory of COMSATS University, Islamabad, Vehari-Campus. Four different doses of each adsorbent were used, i.e., 0.25, 0.5, 0.75 and 1.0 g/100 ml of Pb sub-stock solution. All these doses of weighed adsorbents were added separately in 250 ml conical flasks having 100 ml of 7, 15, 30, 60 and 120 ppm Pb sub-stock solutions. These flasks in duplicate were placed on an orbital shaker and shaked at 300 rpm at room temperature (25 ± 1 °C) for 120 min. At the end, representative samples were taken from flasks using a digital pipette. Samples taken from the flasks were then filtered using whatmann filter paper-42. Filtrates obtained from filtration process were analyzed for Pb concentration using Atomic Absorption Spectrometer (AAS).

### Adsorption analysis

After analyzing Pb concentration from collected samples, the adsorption *Q* (mg/g) of Pb ions was calculated using Eq. (1) as proposed by Edokpayi et al. (2015).

(1) \begin{eqnarray*}Q= \left( \frac{{C}_{i}-{C}_{e}}{W} \right) \times V\end{eqnarray*}

Where, *C*_*i*_ is the initial Pb concentration (ppm), *C*_*e*_ is equilibrium concentration of Pb ions, *W* is the adsorbent weight (g) and *V* represents volume of the aqueous solution (ml).

Thereafter, percentage removal (*R*) of Pb by the adsorbents were calculated using Eq. (2).

(2) \begin{eqnarray*}R= \left( \frac{{C}_{i}-{C}_{f}}{{C}_{i}} \right) \times 100\end{eqnarray*}

Where, *C*_*i*_ and *C*_*f*_ represents the initial and final Pb concentrations (ppm) in aqueous solution, respectively.

### Modeling equilibrium adsorption isotherms

Adsorption isotherms are imperative to elaborate the interaction of solutes with adsorbents (Tan & Hameed, 2010). There are different models employed to predict adsorption isotherm. The most commonly used models for adsorption of contaminant from wastewater are Langmuir and Freundlich adsorption isotherms. The former isotherm model considers biosorption of contaminant (Pb) from aqueous solution as monolayer adsorption on the sites of adsorbents and is described by the equation;

(3) \begin{eqnarray*}{q}_{e}= \frac{{q}_{\mathrm{max}}{K}_{L}{C}_{eq}}{1+{K}_{L}{C}_{eq}} \end{eqnarray*}

Where, *q*_max_ represents the max. adsorption capacity of adsorbents (mg g^−1^), *K*_*L*_ is Langmuir sorption constant (Lmg^−1^), and *C*_*eq*_ is Pb concentration at equilibrium (ppm).

Linear form of Eq. (3) can be represented as:

(4) \begin{eqnarray*} \frac{{C}_{eq}}{{q}_{e}} = \frac{1}{{q}_{\mathrm{max}}{K}_{L}} + \frac{1}{{q}_{\mathrm{max}}} {C}_{eq}\end{eqnarray*}

A linear plot of *C*_*eq*_∕*q*_*e*_ versus *C*_*eq*_ was used to calculate values of *K*_*L*_ and *q*_max_ from its intercept and slope, respectively.

Adsorption on heterogeneous surface of the adsorbents is described by Freundlich isotherm model which assumes multilayer adsorption of the metal due to uneven distribution of sorption energy over the surface of adsorbent (Freundlich, 1906). Mathematically it can be written as:

(5) \begin{eqnarray*}{q}_{e}={K}_{F}{C}_{eq}^{1/n}\end{eqnarray*}

Where, *q*_*e*_ represents the sorption at equilibrium (mg g^−1^), *C*_*eq*_ depicts Pb concentration at equilibrium (ppm), *K*_*F*_ represents the Freundlich sorption constant and *n* is the intensity of adsorption or heterogeneity of the material. Linear form of Eq. (5) can be shown as:

(6) \begin{eqnarray*}\log \nolimits ~{q}_{e}=\log \nolimits ~{K}_{F}+ \frac{1}{n} \log \nolimits ~{C}_{eq}\end{eqnarray*}

The value of *n* and *K*_*F*_ parameters were calculated from slope and the intercept of linear plot between log *q*_*e*_ and log *C*_*eq*_, respectively.

Adsorption and removal of Pb by NB, PB and APB were statistically analyzed using analysis of variance in STATISTIX 8.1. When, the overall main effects were statistical significant, treatments were further compared using Tukey’s test at 5% probability level.

## Results

### Characterization of adsorbents

#### Fourier Transform Infrared Spectra (FTIR)

**Figure 1:**
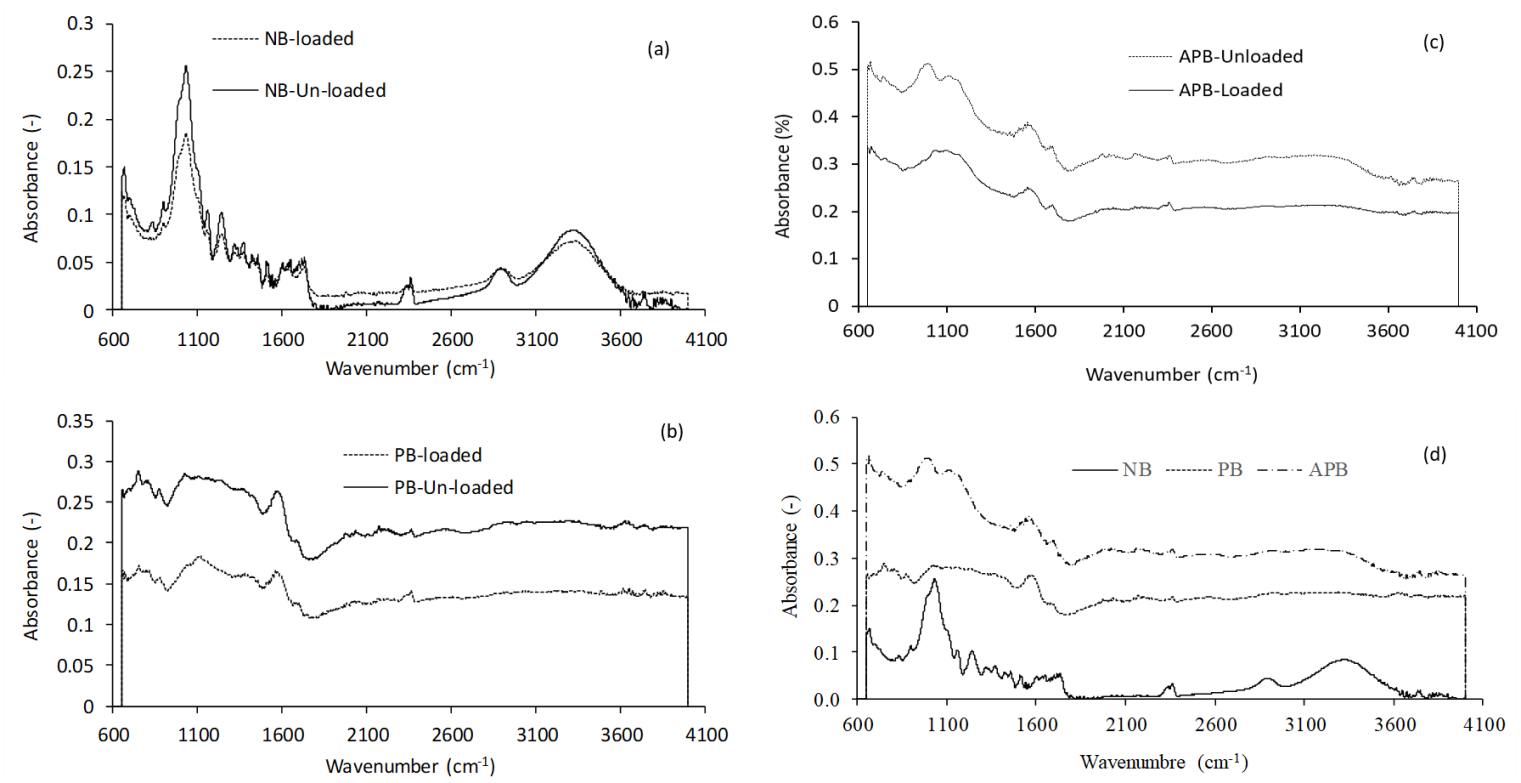
Fourier transform infrared (FTIR) spectra of loaded and unloaded (A) natural sugarcane bagasse (NB), (B) pyrolysed sugarcane bagasse (PB), (C) acid-assisted pyrolysed sugarcane bagasse (APB and (D) unloaded NB, PB, APB.

FTIR spectroscopy is used for identification of different functional groups on adsorbent materials. FTIR spectra of Pb loaded and unloaded natural sugarcane bagasse (NB), pyrolysed sugarcane bagasse (PB) and acid-assisted pyrolysed sugarcane bagasse (APB) are shown in Figs. 1A–1C. Table 1 shows the functional groups assigned to the specific wavenumber. Broad band at 3,332 cm^−1^, 3,334 cm^−1^, 3,250 cm^−1^ and 3,287.27 cm^−1^wavenumber shows the presence of bonded-OH groups. The band observed at 2,893 cm^−1^ represents aliphatic C-H group, whereas sorption peaks at 1,541 and 1,521 cm^−1^ indicate C=O stretching. The one peak observed at 1,273 and 1,276 cm^−1^ corresponds to C-H bending. On the basis of FTIR, observer can confirm the biosorption potential of different adsorbents with sufficient and satisfactory removal efficiency. FTIR spectra indicate that NB and PB exhibit the functional groups as –OH, C=O, and sp^3^ C-H in their chemical structure. All these structures are connected to each other through hydrogen bonding.

**Table 1:** Comparative data of FTIR of the adsorbents used in experiment.

**Peak wavenumber (cm**^−1^**)**							
**NB**		**PB**		**APB**		**Range of IR bands (cm**^−1^**)**	**Functional Group**
Loaded	Unloaded	Loaded	Unloaded	Loaded	Unloaded		
3,332	3,334	3,362	3,371	–	–	3,400–3,300	-OH group
–	–	–	–	2,893	2,993	3,000–2,850	SP^3^ C-H stretching
1,631	1,642	–	–	–	–	1,680–1,630	C=O stretching
1,508	1,540	1,541	1,558	1,558	1,557	1,600–1,450	C=C aromaticity
strong intense at 1,000		strong intense at 1,000	1,273	1,276	strong intense at 1,000	strong intense absorption at 1,300–1,000	C-O ester
Present		Present	Present	Present	Present	Broad bandat 3,400–3,300	-OH hydrogen bonding

### Scanning electron microscopy (SEM)

**Figure 2:**
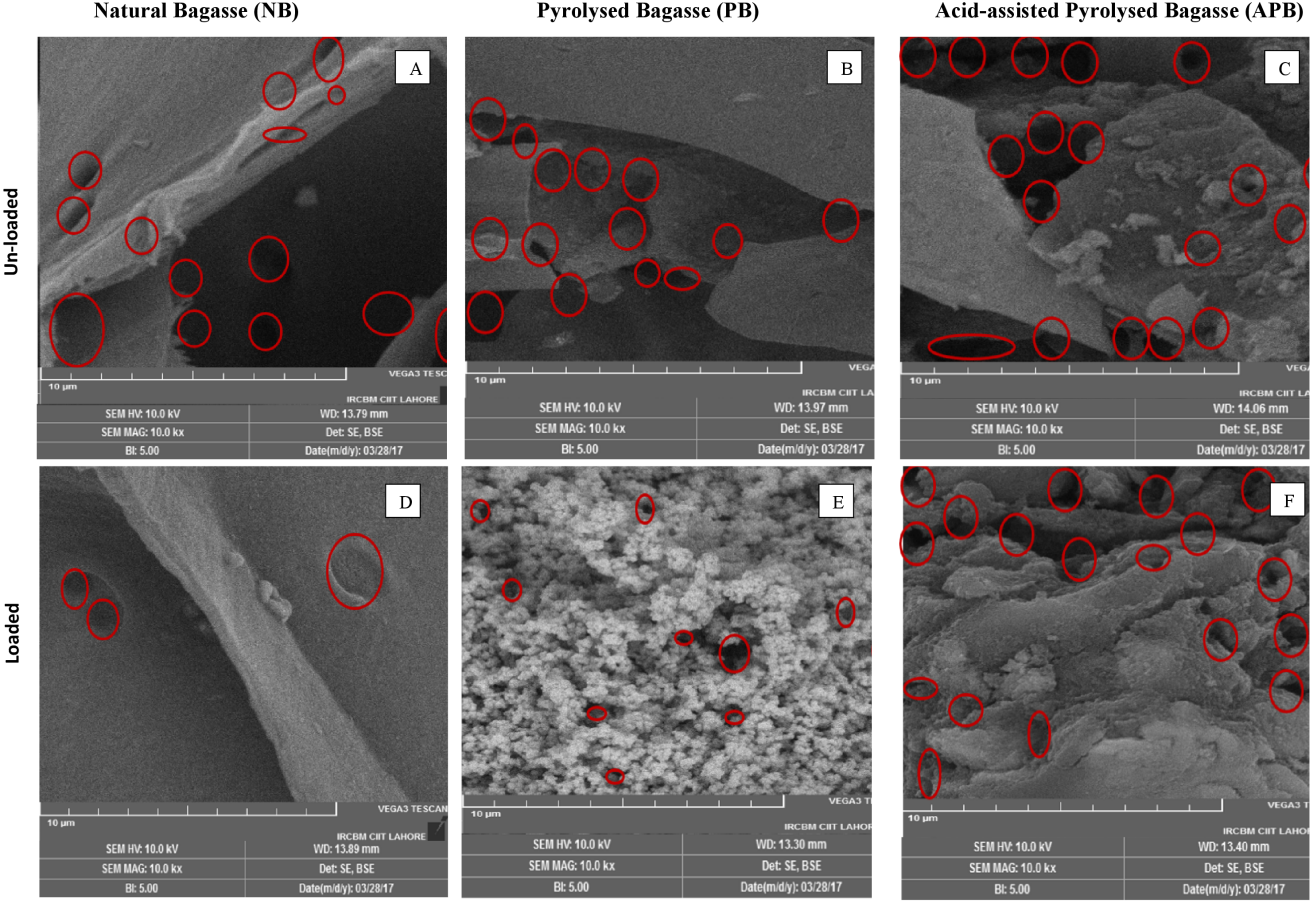
SEM micrograph of un-loaded and loaded NB (A and D, respectively), PB (B and E, respectively), and APB (C and F, respectively) (magnification-10 Kx, SEM HV-10 KV). Circles on images indicate the pores.

The surface morphology of loaded and unloaded NB, PB and APB adsorbents was determined using scanning electron microscope and are shown in Fig. 2. The results illustrated that NB was less porous as compared to PB or APB and the pores were filled, in all probability, with Pb ions as a result of adsorption. PB was relatively less porous as compared with APB, has small particle size with large surface area and well-developed cavities. After adsorption of Pb ions, the surface of PB were changed to microporous structure. APB contained large number of wide pores with rough texture and heterogeneous surface which were converted into small pores after adsorption of Pb ions.

### Effect of adsorbent types and initial Pb concentration

**Figure 3:**
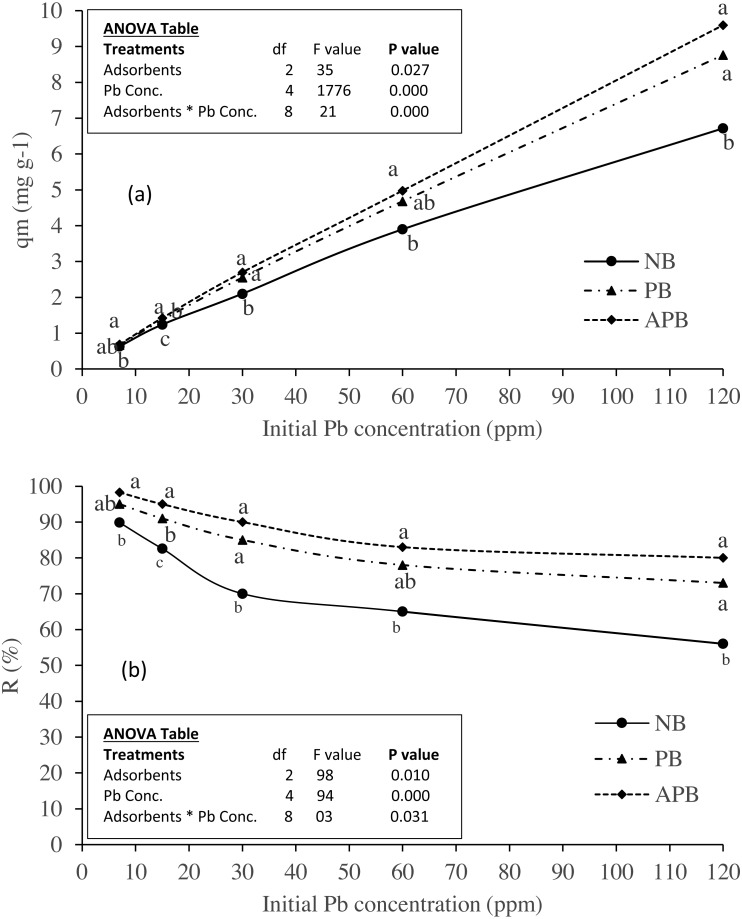
Effect of adsorbent types on (A) Pb adsorption and (B) Pb removal (%) at equilibrium under various levels of Pb contaminated aqueous solution (adsorbent dose one g per 100 ml; contact time 120 minutes).

Effect of different adsorbent types on Pb adsorption and removal (%) at equilibrium under different Pb concentration in aqueous solution are presented in Figs. 3A &  3B, respectively. For both these parameters. we found significant difference among the adsorbents, initial Pb concentrations and their interactions (*P* < 0.05, Figs. 3A & 3B). Results revealed that adsorption *q*_*e*_ of Pb increased with the initial Pb concentration in the aqueous solution, irrespective to the adsorbent type. Among the adsorbent types, APB adsorbed maximum Pb ions at a given initial Pb concentration than NB and PB (*P* < 0.05). At the initial Pb concentration of 7 ppm, there was no statistical difference in adsorption between the NB and PB (0.66 Vs. 0.63 mg g^−1^, *P* > 0.05). However, this difference was pronounced with the increase in initial Pb concentration. At 120 Pb concentrations, *q*_*e*_ values were 6.72, 8.76 and 9.6 mg g^−1^ for NB, PB and APB, respectively. However, from all the adsorbents the maximum removal was achieved at the least Pb initial concentration (7 ppm). At this Pb concentration, NB removed 90% of the Pb ions from solution, whereas the respective removal in case of PB and APB were 95 and 98%, respectively (Fig. 3B). This % Pb removal showed inverse relation with initial Pb concentration. At the highest initial Pb concentration of 120 ppm only about 56% of the initial Pb was removed, whereas this fraction was 73 and 80% in case of PB and APB, respectively.

**Figure 4:**
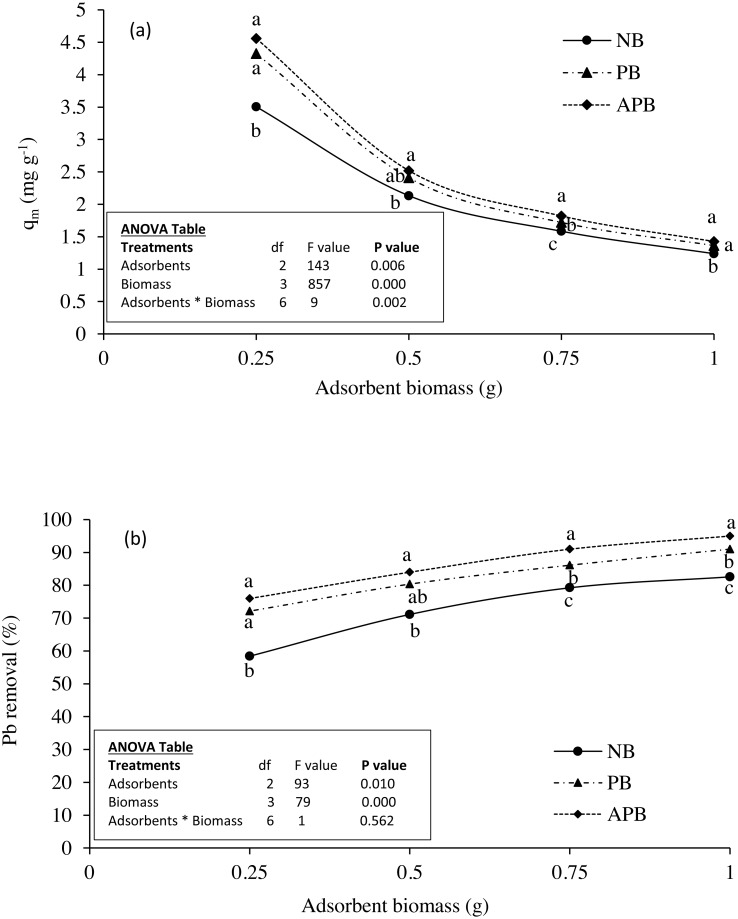
Effect of adsorbent types on (A) Pb adsorption and (B) Pb removal (%) at equilibrium under various doses of the adsorbents in 100 ml Pb contaminated aqueous solution (Pb concentration 15 ppm; contact time 120 min).

### Effect of adsorbent mass

Figures 4A and 4B present Pb adsorption and % removal from contaminated water, respectively. Significant effect of adsorbent biomass on Pb adsorption and removal was found (*P* < 0.05). The highest values of Pb ion adsorbed on each adsorbent were found at mass of 0.25 g per100 ml of contaminated water, which were further decreased with the increase in adsorbent mass. At this biomass, APB adsorbed about 30% higher Pb ions as compared to NB (3.50 vs. 4.56 mg g^−1^ *P* < 0.05). This percent increase was reduced with the increase in adsorbent mass: 2.13 vs. 2.52 (18%) at 0.5 g adsorbent biomass, 1.58 vs. 1.82 (16%) at 0.75 g adsorbent biomass and 1.23 vs. 1.42 (15%) at 1 g adsorbent biomass. Adsorption by PB was also higher than NB material but slightly lower than the APB at each adsorbent biomass.

Among the adsorbent biomass, percentage removal of Pb was the highest at 1 g and least at 0.25 g for each adsorbent type. At this biomass, about 58, 72 and 76% of Pb was removed by NB, PB and APB, respectively. The respective values at 1 g biomass were 83, 91 and 95. Difference between the NB and APB was more pronounced at the least adsorbent biomass (i.e., 0.25 g) which further narrowed down with the increase in adsorbent biomass from 0.25 to 1 g (Fig. 4B). At each adsorbent biomass, % removal followed the order APB>PB>NB (Fig. 4B).

### Equilibrium isotherm models

**Figure 5:**
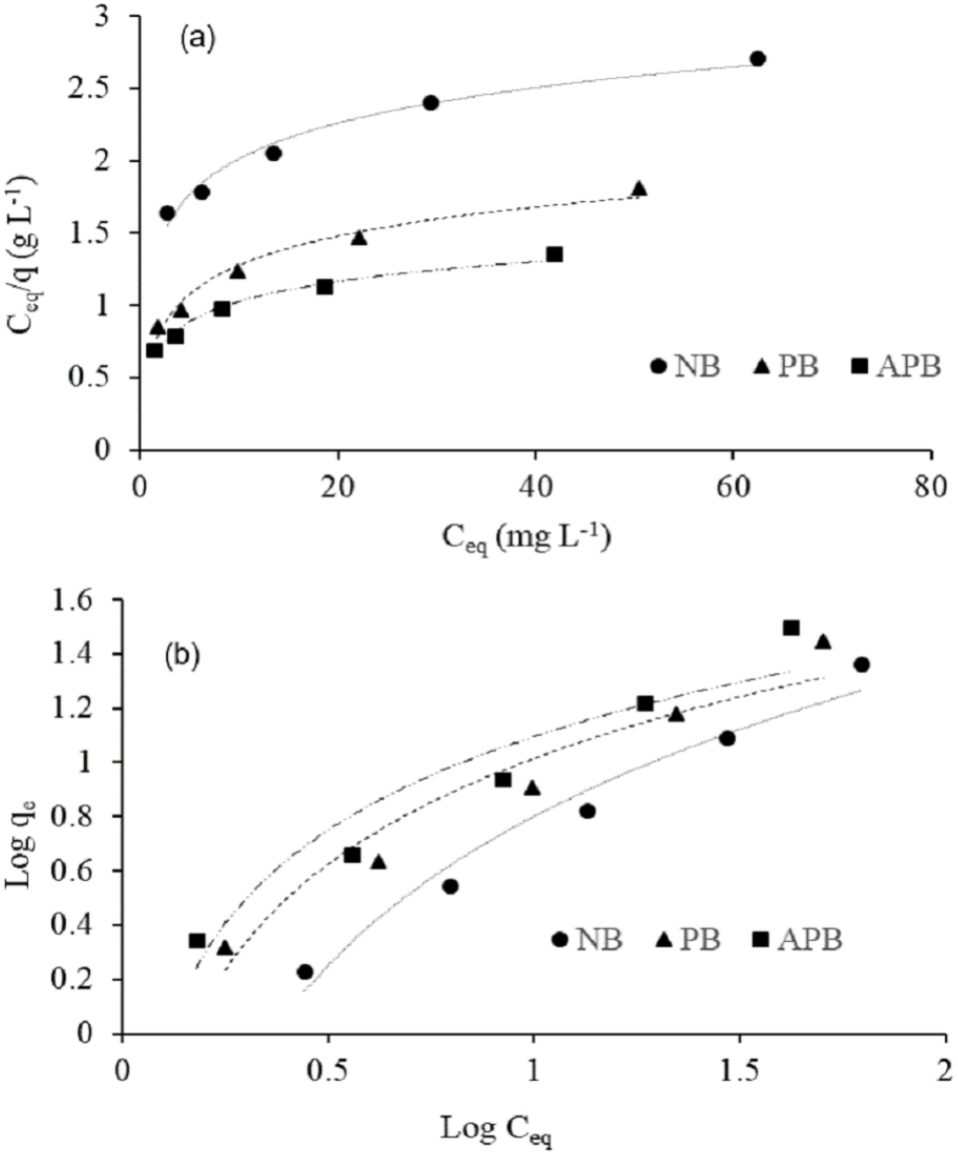
Equilibrium adsorption isotherm models: Langmuir isotherm (A) and Freundlich isotherm (B) models at adsorbent doze of 0.25 g/100 ml contaminated water, pH 6.3 ± 1.5, room temperature 25 °C (±1.5).

Adsorption isotherm models (Eqs. (4) and (6)) explain the equilibrium data for Pb biosorption by NB, PB and APB. Plot of *C*_*eq*_∕*q*_*e*_ and *C*_*eq*_ for Langmuir and plot of log *C*_*eq*_ and log *q*_*e*_ for Freundlich revealed straight lines (Fig. 5). Values of Langmuir and Freundlich parameters were calculated from the intercept and slope of plot (Table 2). Values of *q*_*m*_-calculated from these models reveal that predicted value from Freundlich model is very close to the *q*_*m*_-experimental irrespective to the biosorbent type (Table 2). Further, coefficient of determination showed that Pb ion sorption is well described by Freundlich adsorption isotherm for all the adsorbent types (*R*^2^ = 0.999; Table 2). The respective value of coefficient of determination *R*^2^ in case of Langmuir adsorption isotherm was in the range 0.90–0.92. Whereas, *q*_max_ values were 53.475, 57.803, and 66.225 mg g^−1^ for NB, PB and APB materials, respectively. The respective values for *K*_*L*_ were, 0.0109, 0.019, and 0.199 (Table 2). In case of Freundlich model, values of model parameter *n* were 1.202 for NB, 1.302 for PB and 1.257 for APB material. In case of *K*_*F*_ the values were 0.743, 1.387 and 1.606 for NB, PB and APB, respectively (Table 2).

**Table 2:** Parameters of equilibrium adsorption isotherms for the adsorption of Pb from natural sugarcane bagasse (NB), pyrolysed sugarcane bagasse (PB), and acid assisted pyrolysed bagasse (APB).

**Isotherm Model**	**NB**	**PB**	**APB**
** Langmuir**			
q_m_-experimental (mg g^−1^)	9.415	11.483	12.631
q_m_-calculated (mg g^−1^)	9.482	11.631	9.699
q_max(_mg g^−1^_)_	53.476	57.803	66.225
K_L_	0.011	0.019	0.019
R^2^	0.915	0.920	0.906
**Freundlich**			
q_m_ − calculated (mg g^−1^)	9.441	11.521	12.651
Kf	0.743	1.387	1.606
n	1.202	1.302	1.257
R^2^	0.999	0.999	0.999

## Discussion

### Effect of adsorbent type

Among the adsorbent types, APB adsorbed maximum Pb ions at a given initial Pb concentration as compared with NB and PB (*P* < 0.05). For each adsorbent type, maximum Pb removal occurred at an adsorbent mass of 1.0 g/100 ml of aqueous solution. At this adsorbent mass and 120 ppm Pb concentration, removal of Pb was the highest by APB (95%) and the least in case of NB (83%), whereas, PB lied in between (91%). APB also has much porous surface and rough texture. The pores have longitudinal shape and have capillary tubes (Fig. 2) that might be involved in entrapping Pb ions. Further, surface morphology revealed the existence of pores having various sizes and shapes on the surface of adsorbents that might have favored Pb adsorption as evident from figure showing adsorbed Pb particles. There are apparent changes in unloaded and Pb loaded SEM images. There is less unevenness on the loaded material as a result of Pb adsorption on the cell wall matrix and cross linkages, whereas the unloaded material showed uneven structure, irrespective to the adsorbent type. Further, size of pores decreased after the Pb adsorption, especially in case of PB and APB material. Similar changes in surface properties of the adsorbents after treatment with acid were observed by Rasouli et al. (2014). In case of PB, the large pores were converted into microporous structure as evident in Fig. 2, in all probability due to coverage with Pb ions. Fourier transform infrared (FTIR) spectra indicates that NB, PB, and APB exhibit the functional groups including –OH, C=O, C-O, C=C and sp^3^ C-H in their chemical structure. All these structures are connected to each other through hydrogen bonding. The highest adsorption by APB might be ascribed to more active sites in the form of dipoles for the absorption of Pb. So, it might have more ability to adsorb metals than other agricultural waste. The higher polarity may be based on two functional groups present in APB i.e., carboxyl groups and amino groups.

### Effect of adsorbent mass

By increasing adsorbent mass from 0.25 to 1.0 g, Pb adsorption (mg g^−1^) decreased but removal of Pb increased (Figs. 4A and 4B). This, in all probability, can be ascribed to the greater surface area and number of adsorbent particles with a larger number of active surface sites for the adsorption process (Ghorbani, Eisazadeh & Ghoreyshi, 2012; Ahmad et al., 2017). As for as, the adsorbent types are concerned, the highest Pb removal from aqueous solution was obtained by APB (98%) as compared to NB (85%) and PB (90%) with adsorbent mass of 1.0 g (*P* < 0.05). Results showed that adsorbate removal efficiency increased with the adsorbent mass, however, Pb sorption per unit mass decreased. This might be due to the incomplete use of adsorption sites or aggregation in response to high adsorbent mass. In such aggregations, total surface area of the adsorbent particles decreases as reported in earlier studies (Hanif et al., 2007; Lupean, Bulgariu & Macoveanu, 2012). These results are in line with Ahmad et al. (2017) who also found decrease in Cd adsorption with the increase of adsorbent biomass from 0.25 to 1.0 g per 100 ml of aqueous solution. With the increase in adsorbent mass higher than 0.25 g, the incremental Pb removal becomes very low. The probability may be due to the establishment of an equilibrium between the adsorbents active sites and the solution Pb concentration with each other (Boudrahem, Soualah & Aissani-Benissad, 2011).

### Effect of initial concentration of Pb

Effect of various initial Pb concentrations on its adsorption and removal (%) with different adsorbents is represented in Figs. 3A–3B. Results showed increase in Pb adsorption per gram of adsorbent with the increase in initial Pb concentration in aqueous solution. This can be considered as an electrostatic field exists around the adsorbent particles which causes increase in electrostatic interaction between the absorbate and adsorbent particles (Jiang et al., 2006). For each adsorbent type, the greatest Pb adsorption was observed at an initial Pb concentration of 120 ppm at mass 0.25 g per 100 ml aqueous solution but Pb removal was against to it, i.e., maximum removal was achieved when Pb initial concentration was seven ppm with adsorbent mass of 1.0 g. At 1.0 g of adsorbent mass, the decrease in adsorption capacity, as compared to the 0.25 g dose, could be due to the aggregation of adsorbent particles which decreased the potential adsorption sites (Bulut & Baysal, 2006).

### Effect of equilibrium isotherm models

The equilibrium data for Pb biosorption by NB, PB and APB showed that values of *q*_*m*_-calculated from Langmuir model did not describe properly the relationship between the amount of metal ion adsorbed and their equilibrium concentration in the solution. The values predicted from Freundlich model showed a better fit to the *q*_*m*_-experimental (Fig. 5 and Table 2). Further, coefficient of determination (*R*^2^ = 0.999) showed that Pb ion sorption is well described by Freundlich adsorption isotherm for all the adsorbent types. The respective value of coefficient of determination *R*^2^ in case of Langmuir adsorption isotherm was in the range 0.90–0.92. These evidences reflect that Freundlich model is suitable to describe the biosorption behavior of Pb by NB, PB and APB due to heterogeneous nature of the sorbents with several adsorption energies involved in the sorption process. In Freundlich adsorption isotherm, the *K*_*F*_ indicates the adsorption capacity of the material. The values of 1∕*n* (between 0 and 1) and Freundlich equilibrium constant (*K*_*F*_) showed that aforementioned materials favored Pb sorption under the given conditions. This corroborates with Ahmad et al. (2017) who reported a strong correlation of compost for Cd adsorption on the basis of *K*_*F*_ value. However, biosorption potential of sugarcane bagasse was improved with pyrolysis and acid activation.

Adsorption capacities for Pb (mg g^−1^) by SB have been compared with various other adsorbents as reported in literature (Table 3). There exists a large discrepancies in adsorption capacities among various adsorbents. This might, in all probability, be due to the differences in their chemical compositions, surface morphology, functional groups and particle size (Abdel Salam, Makki & Abdelaal, 2011; Shah et al., 2018; Imran et al., 2018). Results of this study showed that SB has good adsorption capacity and is comparable with some other biosorbents. The observed adsorption capacity of NB in this study is well in line with Putra et al. (2014). SB is a fibrous material and consists of about 23% lignin, 27% polyoses, and 50% cellulose (Martín-Lara et al., 2010). This together with, surface heterogeneity and presence of carbon-oxygen containing functional group makes SB a good adsorbent as compared to the others. It is of worth to note that the adsorption capacity of SB was further increased after pyrolysis and activation with phosphoric acid. This can be ascribed to the modification in the functional groups. According to Wan Ngah & Hanafiah (2008), metal adsorption by the lingo-cellulosic material (i.e., SB) mainly occurs through chemical functional groups (carboxylic, amino and phenolic group) which can be further modified by the acid addition and heat treatment. This might have been the case with PB and APB where the metal sorption was further increased as compared with NB.

**Table 3:** Comparison of sorption capacity (mg g^−1^) of various adsorbents as reported in literature.

**Biomass**	**q (mg g**^−1^**)**	**Reference**
Tomato waste	158	Heraldy et al. (2018)
Apple juice	108	Heraldy et al. (2018)
Mango peel	99	Iqbal, Saeed & Zafar (2009)
Jatoba fruit shell	49	Souza et al. (2017)
Sugar beet pulp	44	Pehlivan et al. (2008)
Activated sugarcane bagasse	31	This study
Pyrolysed sugarcane bagasse	28	This study
Pine cone shells	25	Martín-Lara et al. (2016)
Natural Sugarcane bagasse	23	This study
Natural Sugarcane bagasse	21	Putra et al. (2014)
Apple residues	16	Lee et al. (1998)
*Moringa oleifera* seed powder	15	Adhiambo, Lusweti & Moranga (2015)
Rice husk	11	Chuah et al. (2005)
Grape stock	9	Martinez et al. (2006)
Cedar leaf ash	8	Hafshejani et al. (2015)
H_2_SO_4_-modified Sugarcane bagasse	7	Martín-Lara et al. (2010)
Cocoa pod husk	5	Obike et al. (2018)
Almond shells	2	Mehrasbi et al. (2009)
Maize cob	2	Igwe & Abia (2007)
*Moringa oleifera* seeds	1	Aziz, Jayasuriya & Fan (2016)

## Conclusions

Results revealed that all the tested sorbents have potential to adsorb and remove Pb ions from the contaminated water. In this regard, APB proved more effective since it removed 98% of Pb from contaminated water at initial Pb concentration of seven ppm. The respective values of Pb removal in case of NB, and PB were 90 and 95%, respectively. For a given adsorbent type, Pb adsorption increases with increasing the mass of sorbent from 0.25 to 1.0 g per 100 ml of aqueous solution. However, the highest Pb removal occurred at adsorbent mass of 1.0 g per 100 ml of aqueous solution. Initial Pb concentration had a great impact on Pb adsorption and removal by adsorbent. At seven ppm Pb concentration, maximum Pb removal took place, irrespective to the adsorbent type, whereas, the least in case on 120 ppm Pb concentration. Experimental data showed better agreement with Freundlich adsorption isotherm model. Overall, biosorption potential of the adsorbents was dependent on adsorbent type, adsorbent mass, and initial Pb concentration. However, in order to optimize Pb removal by the above mentioned adsorbent, further studies on the effect of other parameters, i.e., solution pH and temperature need to be investigated.

##  Supplemental Information

10.7717/peerj.5672/supp-1 Data S1 Raw data file

## Additional Information and Declarations

### Data Availability

The following information was supplied regarding data availability: The raw data are provided in Data S1.

## References

[ref-1] Abas SNA, Ismail MHS, Kamal ML, Izhar S (2013). Adsorption process of heavy metals by low cost adsorbent. World Applied Sciences Journal.

[ref-2] Abdel Salam M, Makki MSI, Abdelaal MY (2011). Preparation and characterization of multi-walled carbon nanotubes/chitosan nanocomposite and its application for the removal of heavy metals from aqueous solution. Journal of Alloys and Compounds.

[ref-3] Adhiambo OR, Lusweti KJ, Moranga GZ (2015). Biosorption of Pb2+ and Cr2+ using Moringa Oleifera and their adsorption isotherms. Science Journal of Analytical Chemistry.

[ref-4] Agency for Toxic Substances and Disease Registry (ATSDR) (2008). Toxicology profile for cadmium.

[ref-5] Ahalya N, Kanamadi RD, Ramachandra TV (2005). Biosorption of heavy metals. Electronic Journal of Biotechnology.

[ref-6] Ahmad I, Akhtar MJ, Jadoon IBK, Imran M, Imran M, Ali S (2017). Equilibrium modeling of cadmium biossorption from aqueous solution by compost. Environmental Science and Pollution Research.

[ref-7] Anastopoulos I, Bhatnagar A, Hameed BH, Ok YS, Omirou M (2017). A review on waste-derived adsorbents from sugar industry for pollutant removal in water and wastewater. Journal of Molecular Liquids.

[ref-8] Annadurai G, Juang RS, Lee DJ (2002). Use of cellulose based waste for adsorption of dyes from aqueous solution. Journal of Hazardous Materials.

[ref-9] Asrari E, Tavallali H, Hagshenus M (2010). Removal of Zn (II) and Pb(II) ions using rice husk ih food industrial waste water. Journal of Applied Sciences and Environmental Management.

[ref-10] Atkinson BW, Bux F, Kasan HC (1998). Considerations for application of bio sorption technology to remediate metal-contaminated industrial effluents. Water SA.

[ref-11] Aziz NAA, Jayasuriya N, Fan L (2016). Adsorption study on Moringa oleifera seeds and Musa cavendish as natural water purification agents for removal of Lead, Nickel and Cadmium from drinking water.

[ref-12] Badmus MAO, Audu TOK, Anyata B (2007). Removal of copper badfrom industrial wastewaters by activated carbon prepared from periwinkle shell. Korean Journal of Chemical Engineering.

[ref-13] Bentama J, Schmitz P, Destrac P, Espenan JM (2004). Technological innovation for the production of drinking water by membrane processes. Desalination.

[ref-14] Boudrahem F, Soualah A, Aissani-Benissad F (2011). Pb(II) and Cd(II) removal from aqueous solutions using activated carbon developed from coffee residue activated with phosphoric acid and zinc chloride. Journal of Chemical & Engineering Data.

[ref-15] Bulut Y, Baysal Z (2006). Removal of Pb(II) from wastewater using wheat bran. Journal of Environmental Management.

[ref-16] Charerntanyarak L (1999). Heavy metals removal by chemical coagulation and precipitation. Water Science & Technology.

[ref-17] Chaudhary H, Ijaz M (2014). Removal of lead from wastewater by adsorption on Saponified melon peels gel. Science International.

[ref-18] Chen CX, Huang B, Li T, Wu GF (2012). Preparation of phosphoric acid activated carbon from sugarcane bagasse by mechanochemical processing. BioResources.

[ref-19] Cheremisinoff PN, Morresi AC (1978). Carbon adsorption handbook.

[ref-20] Chuah TC, Jumasiah A, Azni I, Katayan S, Choong SYT (2005). Rice husk as a potentially lowcost biosorbent for heavy metal and dye removal: an overview. Desalination.

[ref-21] Dabrowski A, Hubicki Z, Podkościelny P, Robens E (2004). Selective removal of the heavy metal ions from waters and industrial wastewaters by ion-exchange method. Chemosphere.

[ref-22] Demirbas A (2008). Heavy metal adsorption onto agro-based waste materials: a review. Journal of Hazardous Materials.

[ref-23] Edokpayi JN, Odiyo JO, Msagati TAM, Popoola EO (2015). A novel approach for removal of Lead (II) ions from wastewater using Mucilaginious leaves of Diceriocaryum Eriocarpum plant. Sustainability.

[ref-24] El-Said AG, Gamal AM, Mansour HF (2012). Potential application of Orange peel (OP) as an eco-friendly adsorbent for textile dyeing effluents. Journal of Textile and Apparel, Technology and Management.

[ref-25] Freundlich HMF (1906). Over the adsorption in solution. Journal of Physical Chemistry.

[ref-26] Ghorbani M, Eisazadeh H, Ghoreyshi A (2012). Removal of zinc ions from aqueous solution using polyaniline nanocomposite coated on rice husk. Iranica Journal of Energy and Environment.

[ref-27] Hafshejani LD, Nasab SB, Gholami RM, Moradzadeh M, Izadpanaha Z, SB Hafshejani, Bhatnagar A (2015). Removal of zinc and lead from aqueous solution by nanostructured cedar leaf ash as biosorbent. Journal of Molecular Liquids.

[ref-28] Hanif MA, Nadeem R, Bhatti HN, Ahmad NR, Ansari TM (2007). Ni (II) bio sorption by Cassia fistula (Golden Shower) biomass. Journal of Hazardous Materials.

[ref-29] Heraldy E, Lestari WW, Permatasaria D, Arimurtia DD (2018). Biosorbent from tomato waste and apple juice residue for lead removal. Journal of Environmental Chemical Engineering.

[ref-30] Huang CP, Wu MH (1975). Chromium removal by carbon adsorption. Water Pollution Control Federation Journal.

[ref-31] Hussain AZ, Shariff KMM (2014). Removal of heavy metals from wastewater using low cost adsorbents. Archives of Applied Science Research.

[ref-32] Igwe JC, Abia AA (2007). Adsorption isotherm studies of Cd (II), Pb (II) and Zn (II) ions bioremediation from aqueous solution using unmodified and EDTA-modified maize cob. Eclectica Quimica.

[ref-33] Imran M, Suddique M, Shah GM, Ahmad I, Murtaza B, Shah NS, Mubeen M. Ahmad S, Zakir A, Schotting RJ (2018). Kinetic and equilibrium studies for cadmium biosorption from contaminated water using Cassia fistula biomass. International Journal of Environmental Science and Technology.

[ref-34] Iqbal M, Saeed A, Zafar SI (2009). FTIR spectrophotometry, kinetics and adsorption isotherms modeling, ion exchange, and EDX analysis for understanding the mechanism of Cd2+ and Pb2+ removal by mango peel waste. Journal of Hazardous Materials.

[ref-35] Jiang K, Sun TH, Sun LN, Li HB (2006). Adsorption characteristics of copper, lead, zinc and cadmium ions by tourmaline. Journal of Environmental Sciences.

[ref-36] Kadirvelu K, Thamaraiselvi K, Namasivayam C (2001). Removal of heavy metal from industrial wastewaters by adsorption onto activated carbon prepared from an agricultural solid waste. Bioresource Technology.

[ref-37] Kocasoy J, Guvener Z (2009). Efficiency of compost in the removal of heavy metals from the industrial wastewater. Environmental Geology.

[ref-38] Kong W, Ren J, Wang S, Chen Q (2014). Removal of heavy metals from aqueous solutions using acrylic-modified sugarcane bagasse-based adsorbents: equilibrium and kinetic studies. Bioresource Technology.

[ref-39] Kumar U, Bandyopadhyay M (2006). Sorption of cadmium from aqueous solution using pretreated rice husk. Bioresource Technology.

[ref-40] Lee SH, Jung CH, Chung H, Lee MY, Yang JW (1998). Removal of heavy metals from aqueous solution by apple residues. Process Biochemistry.

[ref-41] Lupean M, Bulgariu L, Macoveanu M (2012). Bio sorption of Cd(II) from aqueous solution on marine green algae biomass. Environmental Engineering and Management Journal.

[ref-42] Martín-Lara MA, Blázquez G, Calero M, Almendros AI, Ronda A (2016). Binary biosorption of copper and lead onto pine cone shell in batch reactors and in fixed bed columns. International Journal of Mineral Processing.

[ref-43] Martín-Lara MÁ, Rico ILR, Vicente IDLCA, García GB, De Hoces MC (2010). Modification of the sorptive characteristics of sugarcane bagasse for removing lead from aqueous solutions. Desalination.

[ref-44] Martinez M, Miralles N, Hidalgo S, Fiol N, Villaescusa I (2006). Removal of lead (II) and cadmium (II) from aqueous solutions using grape stalk waste. Journal of Hazardous Materials.

[ref-45] Mehrasbi MR, Farahmandkia Z, Taghibeigloo B, Taromi A (2009). Adsorption of lead and cadmium from aqueous solution by using almond shells. Water, Air, and Soil Pollution.

[ref-46] Meunier N, Laroulandie J, Blais JF, Dayal TR (2003). Lead removal from acidic solutions by sorption on cocoa shells: effect of some parameters. Journal of Environmental Chemical Engineering.

[ref-47] Milhajlovic MT, Lazarevic SS, Jankovic-Castvan IM, Kovac J, Jokic BM, Janackovic DT, Petrovic RD (2015). Kinetics, thermodynamics, and structural investigations on the removal of Pb2+, Cd2+, and Zn2+ from multicomponent solutions onto natural and Fe (III)-modified zeolites. Clean Technologies and Environmental Policy.

[ref-48] Naeem MA, Khalid M, Aon M, Abbas G, Tahir M, Amjad M, Murtaza B, Yang A, Akhtar SS (2017). Effect of wheat and rice straw biochar produced at different temperatures on maize growth and nutrient dynamics of a calcareous soil. Archives of Agronomy and Soil Science.

[ref-49] Naseem R, Tahir SS (2001). Removal of Pb(II) from aqueous solution by using bentonite as an adsorbent. Water Research.

[ref-50] Obike AI, Igwe JC, Emeruwa CN, Uwakwe KJ (2018). Equilibrium and kinetic studies of Cu (II), Cd (II), Pb (II) and Fe (II) Adsorption from aqueous solution using Cocoa (Theobroma cacao) pod husk. Journal of Applied Sciences and Environmental Management.

[ref-51] Palin Jr D, Rufato K, Linde G, Colauto N, Caetano J, Alberton O, Jesus D, Dragunski D (2016). Evaluation of Pb (II) biosorption utilizing sugarcane bagasse colonized by basidiomycetes. Environmental Monitoring and Assessment.

[ref-52] Pehlivan E, Yanık B, Ahmetli G, Pehlivan M (2008). Equilibrium isotherm studies for the uptake of cadmium and lead ions onto sugar beet pulp. Bioresource Technology.

[ref-53] Putra WP, Kamari A, Mohd Yusoff SN, Ishak CF, Mohamed A, Hashim N, Isa IM (2014). Biosorption of Cu(II), Pb(II) and Zn(II) ions from aqueous solutions using selected waste materials: adsorption and characterization studies. Journal of Encapsulation and Adsorption Sciences.

[ref-54] Rasouli F, Aber S, Salari D, Khataee AR (2014). Optimized removal of reactive navy blue SP-BR by organo-montmorillonite based adsorbents through central composite design. Applied Clay Science.

[ref-55] Renge VC, Khedkar SV, Pande SV (2012). Removal of heavy metals from wastewater using low cost adsorbents. Scientific Reviews and Chemical Communications.

[ref-56] Shah GM, Umm-e aiman, Imran M, Bakhat HF, Hammad HM, Ahmad I, Rabbani F, Khan ZUH (2018). Kinetics and equilibrium study of lead bio-sorption from contaminated water by compost and biogas residues. International Journal of Environmental Science and Technology.

[ref-57] Souza IPAF, Cazetta AL, Pezoti O, Almeida VC (2017). Preparation of biosorbents from the Jatoba (Hymenaea courbaril) fruit shell for removal of Pb (II) and Cd (II) from aqueous solution. Environmental Monitoring and Assessment.

[ref-58] Tan IAW, Hameed BH (2010). Adsorption isotherm, kinetics, thermodynamics and desorption studies of basic dye on activated carbon derived from oil palm empty bunch. Journal of Applied Sciences.

[ref-59] Vasconcelos LAT, Beca CGG (2006). Adsorption equilibria between pine bark and several ions in aqueous solution, 1. Pb(II). European Water Pollution Control.

[ref-60] Wan Ngah WS, Hanafiah MAKM (2008). Removal of heavy metal ions from wastewater by chemically modified plant wastes as adsorbents: a review. Bioresource Technology.

